# Two new species of the genus Anisomysis (Anisomysis) (Crustacea, Mysida, Mysidae) from coral reef waters in Thailand

**DOI:** 10.3897/zookeys.525.5958

**Published:** 2015-10-06

**Authors:** Mitsuyasu Moriya, Khwanruan Srinui, Shozo Sawamoto

**Affiliations:** 1 Atmosphere and Ocean Research Institute, University of Tokyo, 5-1-5 Kashiwanoha, Kashiwa, Chiba 277-8564, Japan; 2 Institute of Marine Science, Burapha University, Muang, Chonburi 20131, Thailand; 3 Department of Marine Biology, School of Marine Science and Technology, Tokai University 3-20-1, Orido, Shimizu, Shizuoka 424-8610, Japan

**Keywords:** *Anisomysis*, Chantaburi, new species, Phuket, taxonomy

## Abstract

Two new species of *Anisomysis* Hansen, 1910 (Mysida, Mysidae), Anisomysis (Anisomysis) spinaintus **sp. n.** and Anisomysis (Anisomysis) phuketensis **sp. n.**, from coral-reef waters in Thailand are described. Anisomysis (Anisomysis) spinaintus, collected in the Chaolao Beach, Chanthaburi Province, is distinguished from the closely allied species Anisomysis (Anisomysis) incisa Tattersall, 1936, and Anisomysis (Anisomysis) hawaiiensis Murano, 1995, by the presence of 6–9 spines on the apical cleft of telson, which are absent in the latter two allied species. The new species can also be distinguished from Anisomysis (Anisomysis) aikawai Ii, 1964, by the presence of a deep telson cleft and a large number of spines on the lateral margin of telson. Anisomysis (Anisomysis) phuketensis **sp. n.**, collected in Ko Lon, Phuket, is distinguished from the allied species Anisomysis (Anisomysis) robustispina Panampunnayil, 1984, by having a short telson and a pair of long spines on the apical part of the telson. Keys to the subgenera and species of *Anisomysis*, including the two new species, are presented.

## Introduction

The genus *Anisomysis* was established by Hansen (1910) to describe *Anisomysis laticauda* collected form Laiwui, Obi Island, Indonesia, during the Siboga Expedition.

Băcescu (1973) divided the genus into two subgenera, *Paranisomysis* and *Anisomysis*, mainly on the basis of the structure of mandibular palp: the subgenus *Paranisomysis* has flagellate tubercles on the inner margin of the second segment of palp, while such tubercles are lacking in the subgenus *Anisomysis*. Furthermore, Băcescu (1992) provisionally created the subgenus *Javanisomysis*, which is characterized by the non-segmented exopod and no endopod of the fourth male pleopod. As the peculiar morphological characteristics were different from the existent characteristics of the genus *Anisomysis*, Murano and Fukuoka (2003) proposed to establish a new genus *Javanisomysis*. The genus *Javanisomysis* is cited as a valid name (i.e. Wittmann et al. 2014), however, recently the genus is re-defined as a subgenus in the genus *Anisomysis* (Sawamoto, Srinui & Moriya, 2015) on the basis of examination of the paratypes of *Javanisomysis gutzui*.

Murano and Fukuoka (2003) carried out a systematic study of the genus *Anisomysis* and created the fourth subgenus, *Pseudanisomysis*, to accommodate a few species that have the eye divided into two parts by a groove based on *Anisomysis bipartoculata*. The genus *Anisomysis* is composed of the four subgenera, *Anisomysis*, *Paranisomysis*, *Pseudanisomysis* (Murano & Fukuoka, 2003) and *Javanisomysis* (Sawamoto et al., 2015), and most of which are known in tropical and subtropical waters of the Indian Ocean, the western and the central Pacific Ocean, and the marginal seas of these oceans (Murano and Fukuoka 2003). According to Mees (2015) the subgenus *Pseudanisomysis* is accepted as a junior synonym of the genus *Carnegieomysis*. However, the latter is insufficiently described by Tattersall (1943) and is re-defined correctly and is housed in the genus *Anisomysis* by Murano (1995).

Currently, the genus Anisomysis contains 36 nominal species in the subgenus Anisomysis, 18 species in the subgenus *Paranisomysis*, and four species in the subgenus *Pseudanisomysis* (Mees, 2015) and three species in the subgenus *Javanisomysis* (Sawamoto et al., 2015). In particular, 15 species in the four subgenera are currently reported from Southeast Asian waters (Sawamoto 2014; Sawamoto et al. 2015). Anisomysis (Anisomysis) thurneysseni is included in the subgenus *Javanisomysis*, but is excluded from the species list of the Southeast Asia. The other two species in the subgenus is added to the list (Sawamoto et al. 2015).

The present paper reports two new species of the subgenus *Anisomysis*, which were discovered during a study of the mysid diversity in Southeast Asia. Keys to the four subgenera and to the 38 species of the subgenus *Anisomysis* have been provided.

## Materials and methods

### Sample collection and morphological measurements

Mysid specimens were collected with a hand net by skin diving in a coral reef in Thailand (see “Systematics” section for details). The mysids from the net samples were immediately fixed in 5% seawater-buffered formalin for morphological analysis and 99% ethanol for genetic analysis, the results of which will be reported elsewhere.

Terminology was mainly based on Murano and Fukuoka (2003). The body length (BL) of the specimens was measured from the anterior end of rostrum to the posterior end of telson as the body was stretched. Illustrations were made with the aid of a camera lucida.

Type specimens are housed in the National Museum of Nature and Science, Japan (NSMT).

## Systematics

### Order Mysida Boas, 1883 Family Mysidae Haworth, 1825 Subfamily Mysinae Haworth, 1825 Tribus Anisomysini Wittmann, Ariani & Lagardère, 2014 Genus *Anisomysis* Hansen, 1910 Subgenus *Anisomysis* Băcescu, 1973

#### Anisomysis (Anisomysis) spinaintus sp. n.

Taxon classificationAnimaliaMysidaMysidae

http://zoobank.org/D61A9F38-853F-40D4-8EA4-00322F37D952

Figs 1 , 2 , 3 , 4

##### Type series.

Holotype (NSMT-Cr 24246), adult male (BL, 4.1 mm); allotype (NSMT-Cr 24247), adult female with embryos (BL, 4.5 mm); paratypes (NSMT-Cr 24248), 3 adult males (BL, 5.0, 4.8 and 4.2 mm) and 2 adult females with embryos (BL, 4.2 and 4.7 mm); Chaolao Beach, Chantaburi Province, Thailand, 12°31.58'N, 101°55.21'E; collected with a hand net (mesh size, 0.33 mm; mouth diameter, 30 cm) by skin diving on a coral reef 3–5 m deep on November 28, 2010 by M. Moriya.

##### Description.

**Figure 1.**
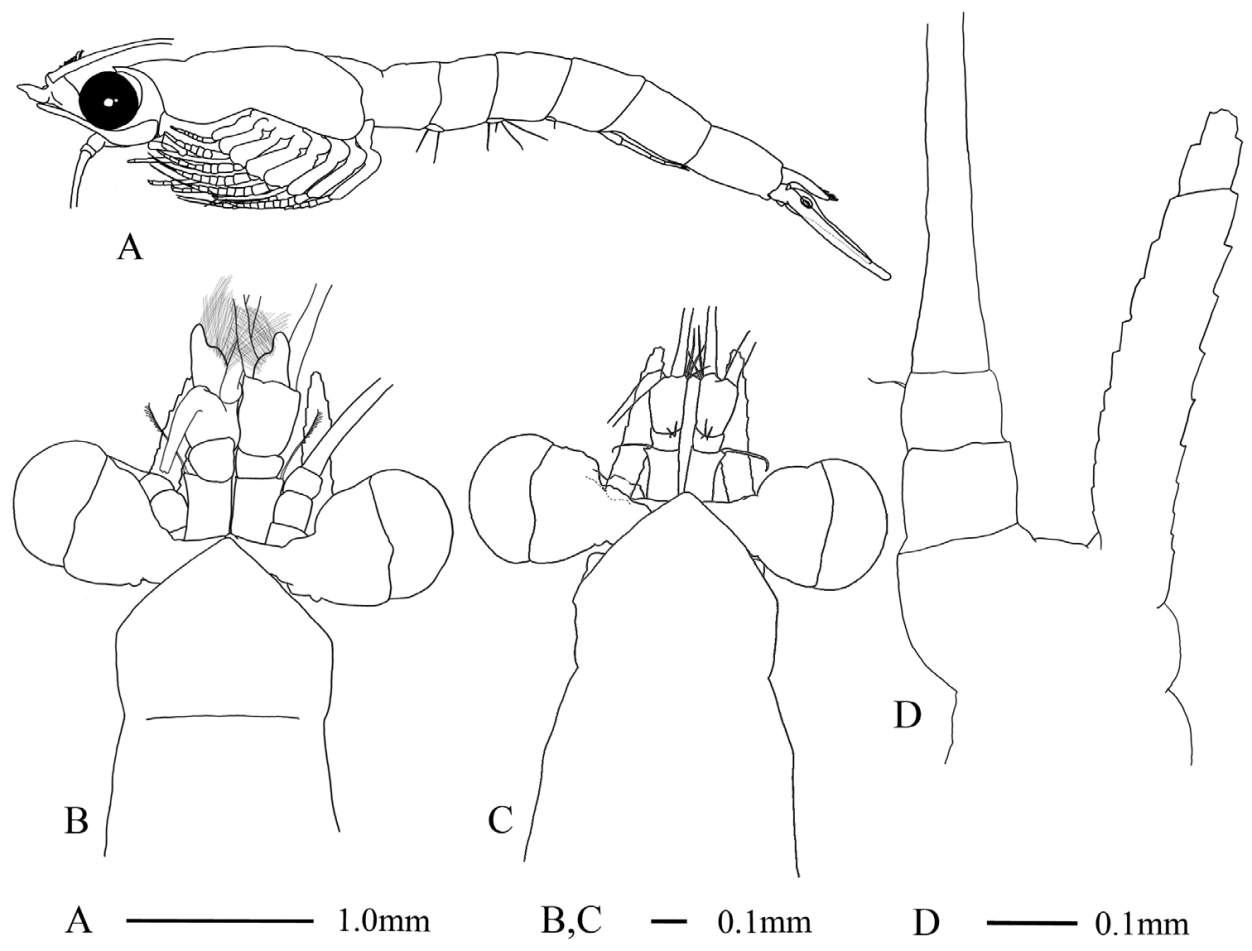
Anisomysis (Anisomysis) spinaintus sp. n., **A, B, D** holotype **C** allotype **A** lateral view **B** anterior part of body **C** anterior part of body **D** antenna.

Body slender (Fig. 1A). Carapace produced anteriorly as a low triangular rostrum with a moderately pointed apex, uncovering eyestalks almost completely (Fig. 1B).

Eyes large, cornea globular, extending laterally beyond the lateral margin of carapace (Fig. 1B, C).

Antennular peduncle more robust in male (Fig. 1B) than that of female (Fig. 1C), first segment as long as third, armed with single seta at anterolateral corner, second segment shortest. In female (Fig. 1C), first segment armed with single seta at anterolateral corner, third segment as long as combined length of first and second segments.

Antennal scale slightly beyond anterior margin of antennular peduncle in male (Fig. 1B), and beyond anterior margin in female (Fig. 1C); 5.9 times as long as broad, slightly curved outward in male (Fig. 1D), 6.7 times as long as broad in female. Antennal peduncle (Fig. 1B, C) short, not reaching the middle of antennal scale in both sexes.

**Figure 2.**
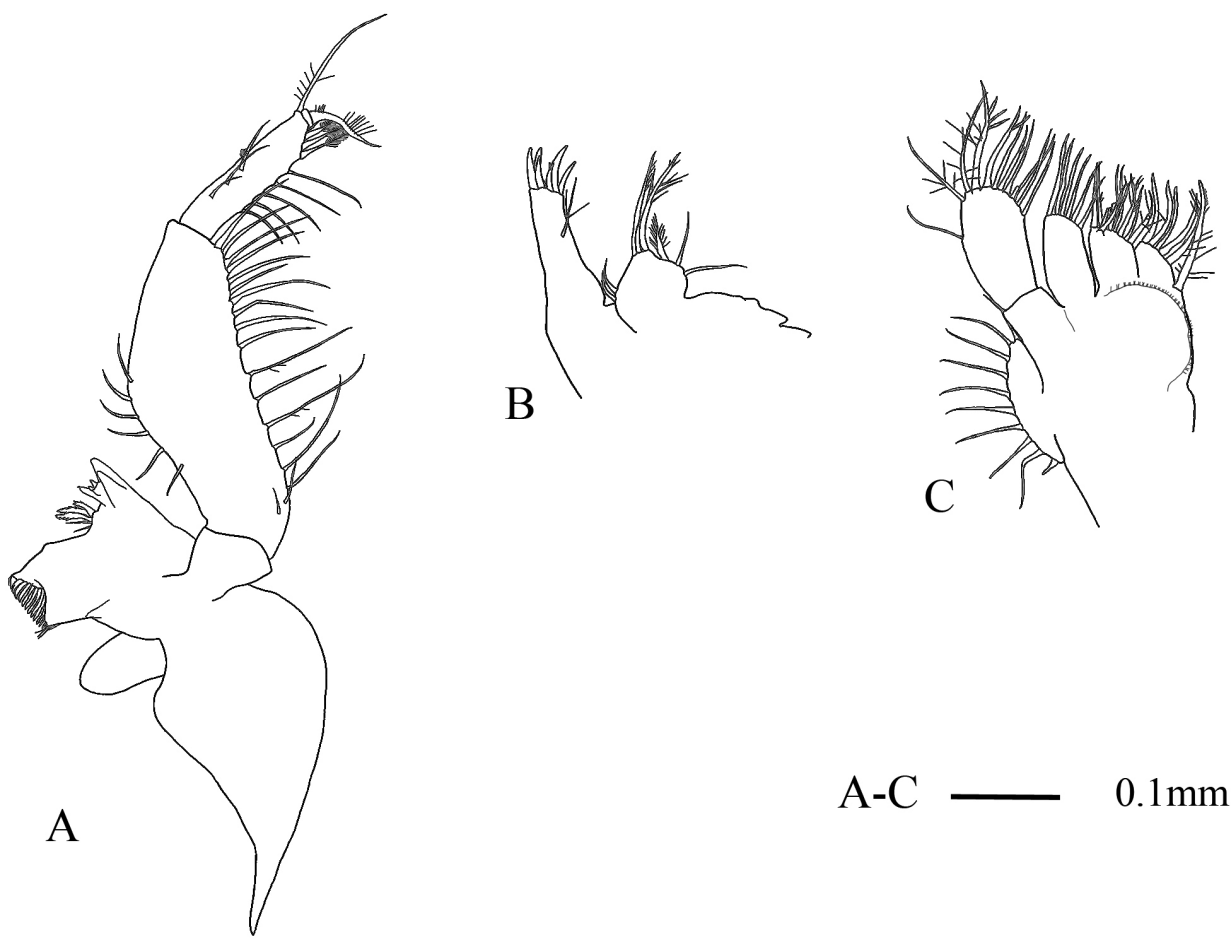
Anisomysis (Anisomysis) spinaintus sp. n., **A–C** holotype; **A** mandible and mandibular palp (right side) **B** maxillule **C** maxilla.

Mandibular palp (Fig. 2A) 3-segmented; second segment widened mesially at around mid-length, armed with setae on both margins, without prominent denticles; third segment 0.6 times as long as second, rectangular, armed with five setae on margin increasing in length distally, with four barbed setae on distal margin and one recurved and barbed seta and 1 long seta at distomedial corner. Maxillule and maxilla as shown in Fig. 2B and C, respectively.

**Figure 3.**
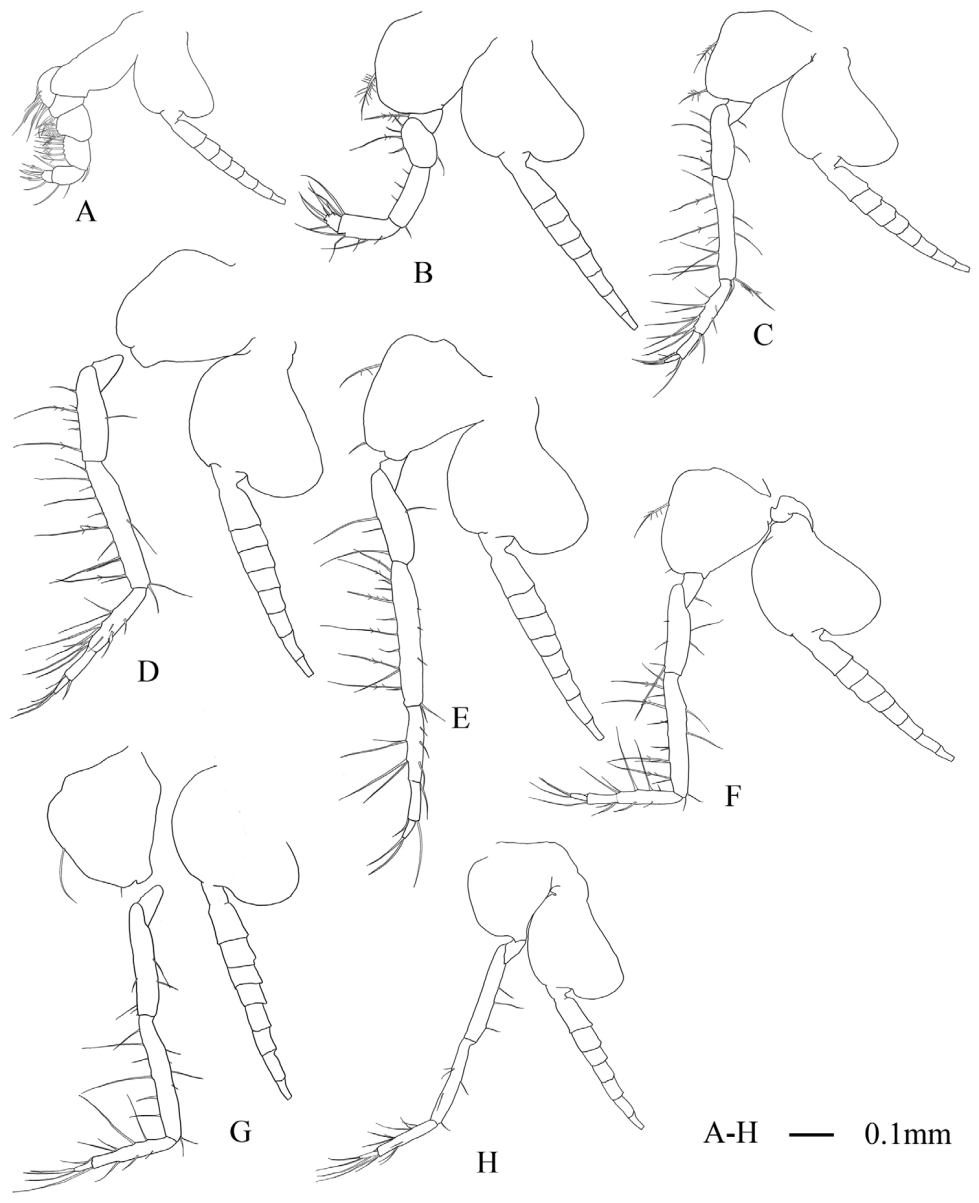
Anisomysis (Anisomysis) spinaintus sp. n., **A–H** holotype **A** 1^st^ thoracopod **B** 2^nd^ thoracopod **C** 3^rd^ thoracopod **D** 4^th^ thoracopod **E** 5^th^ thoracopod **F** 6^th^ thoracopod **G** 7^th^ thoracopod **H** 8^th^ thoracopod.

First thoracopodal endopod (Fig. 3A) short and robust, armed with straight, strong terminal claw. Second thoracopodal endopod (Fig. 3B) short; merus as long as carpopropodus, dactylus slightly longer than broad. Third to sixth thoracopodal endopods (Fig. 3C–F) with carpopropodus divided distally into 2 segments, seventh and eighth thoracopodal endopods (Fig. 3G, H) with undivided carpopropodus in both sexes. Flagelliform part of first and eighth thoracopodal exopods 7-segmented (Fig. 3A, H) and second to seventh 8-segmented (Fig. 3B–G). Basal plates of eight thoracopodal exopods with rounded outer distal corner.

Abdomen (Fig. 1A) long and slender, sixth somite almost as long as fifth.

**Figure 4.**
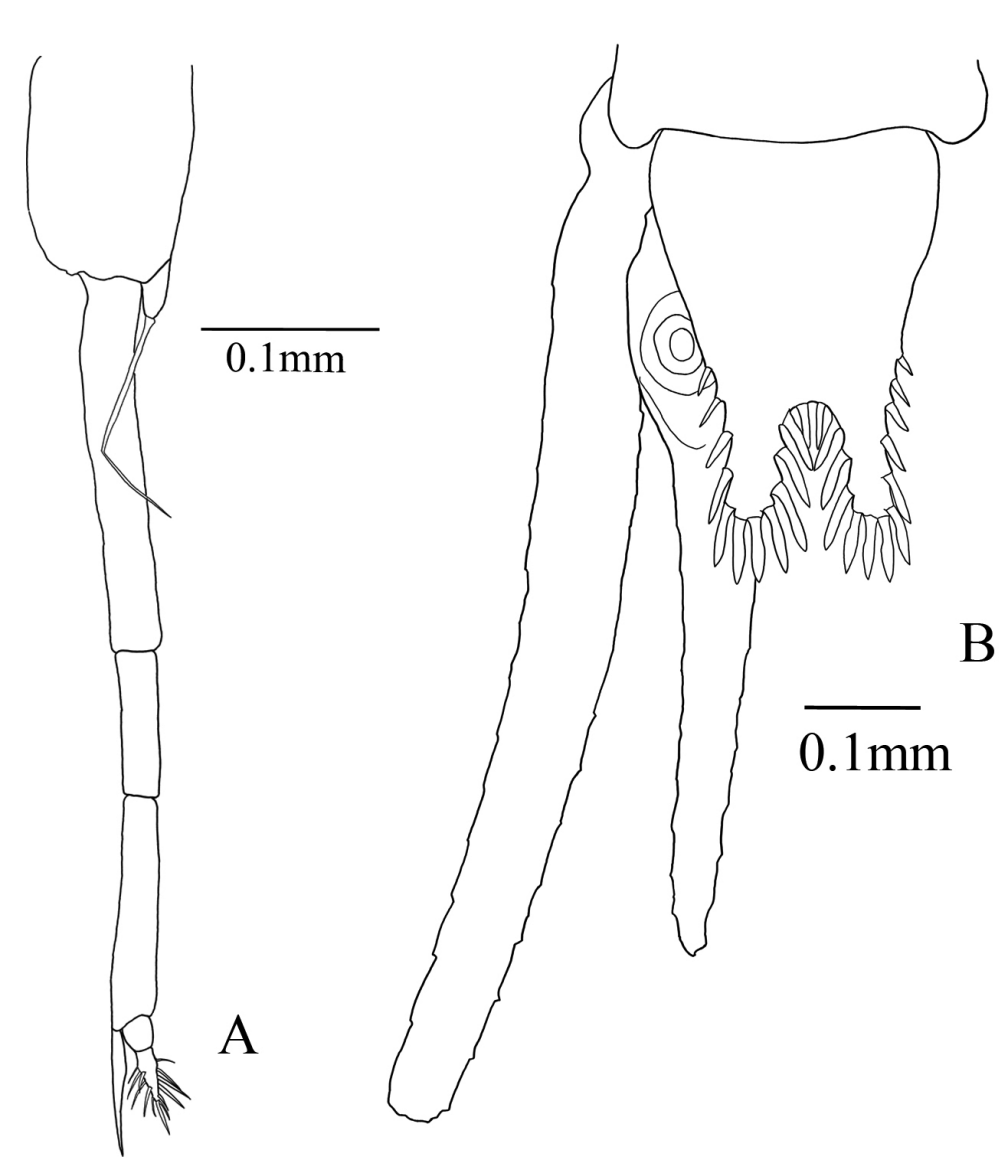
Anisomysis (Anisomysis) spinaintus sp. n., **A, B** holotype **A** 4^th^ pleopod **B** uropods and telson.

First, second, third, and fifth pleopods of males and all pleopods of females rudimentary. Fourth male pleopod (Fig. 4A) biramous; endopod thin lobed with 1 seta, exopod long, 3-segmented, extended to anterior margin of sixth abdominal somite including terminal setae (Fig. 1A); First segment as long as second and third segments combined; second segment shortest; segment length ratios 2.6:1:1.6; third segment with two terminal setae, inner seta slightly shorter than outer, and stout and swollen in proximal part and barbed in distal part, outer seta slender and naked.

Uropod (Fig. 4B) slender, setose; endopod straight, 1.8 times longer than telson excluding apical spines, no spine in statocyst region; exopod slightly curved outward, 1.2 times as long as endopod.

Telson (Fig. 4B) nearly 3/4 length of sixth abdominal somite, 1.7 times as long as broad at base, narrower distally, with apical cleft; lateral margin armed on distal half with 4-7 spines increasing in length distally; distal margin of each apical lobe armed with 2-4 large subequal spines. Apical cleft 2/5 length of telson, slightly more than 1/2 as broad as base at level of cleft, with rounded bottom and 6-9 spines.

##### Etymology.

The specific name is derived from Latin *spina intus*, meaning spine on the inside, referring to the apical cleft of telson armed with spines.

##### Remarks.

The most noticeable characteristic of Anisomysis (Anisomysis) spinaintus is the presence of 6–9 spines on the apical cleft of telson. This new species resembles Anisomysis (Anisomysis) incisa Tattersall, 1936; Anisomysis (Anisomysis) hawaiiensis Murano, 1995; and Anisomysis (Anisomysis) aikawai Ii, 1964, which was re-described by Murano and Fukuoka (2003) on the basis of the specimens from Nomo, Nagasaki, Japan, by the form of the apical cleft of telson. The present species is distinguished from Anisomysis (Anisomysis) incisa and Anisomysis (Anisomysis) hawaiiensis by the presence of spines on the apical cleft of the telson, and from Anisomysis (Anisomysis) aikawai by the deeper apical cleft and larger number of spines on the telson. Differences among these four species are summarized in Table 1.

**Table 1.** Morphological differences among Anisomysis (Anisomysis) spinaintus n. sp; Anisomysis (Anisomysis) incisa Tattersall, 1936; Anisomysis (Anisomysis) hawaiiensis Murano, 1995, Anisomysis (Anisomysis) aikawai Ii, 1964; and Anisomysis (Anisomysis) aikawai Ii, 1964, re-described by Murano and Fukuoka (2003).

	Anisomysis (Anisomysis) spinaintus sp. n.	Anisomysis (Anisomysis) incisa	Anisomysis (Anisomysis) hawaiiensis	Anisomysis (Anisomysis) aikawai
Carpopropodus of 3^rd^ to 8^th^ thoracopodal endopod	3^rd ^to 6^th^ divided distally into 2 segments	Unsegmented	Unsegmented	8^th^ divided distally into 2 segments (at least)
Exopod of 4^th^ male pleopod: length	Anterior margin of 6^th^ abdominal somite	Backwards to level of the apical lobes of the telson	Middle of telson	Backwards to the posterior end of the 5^th^ abdominal somite
Telson: apical cleft	Deep	Deep	Deep	Deep
Spines on each lateral margin of telson	12 or 13	9 or 10	10	11 or 12 (9 or 10) #
Spines on each lateral margin of telson cleft	8	0 (un-armed)	0 (un-armed)	6 (4) #

# Re-described by Murano and Fukuoka (2003).

##### Distribution.

Only known from the type locality.

#### Anisomysis (Anisomysis) phuketensis sp. n.

Taxon classificationAnimaliaMysidaMysidae

http://zoobank.org/1C987A5B-8D8F-436B-A3C3-5B1739089E97

Figs 5 , 6 , 7 , 8

##### Type series.

Holotype (NSMT-Cr 24249), adult male (BL, 3.6 mm); allotype (NSMT-Cr 24250), adult female with embryos (BL, 3.9 mm); paratypes (NSMT-Cr 24251), 2 adult males (BL, 3.8, 4.0 mm) and 2 adult females with embryos (BL, 3.2, 3.6, 3.4 mm); Ko Lon, Phuket Is., Thailand, 7°47.01'N, 98°21.30'E; collected with a hand net (mesh size, 0.33 mm; mouth diameter, 30 cm) by skin diving in a coral reef of 2-3 m deep, December 3, 2010 by M. Moriya.

##### Description.

**Figure 5.**
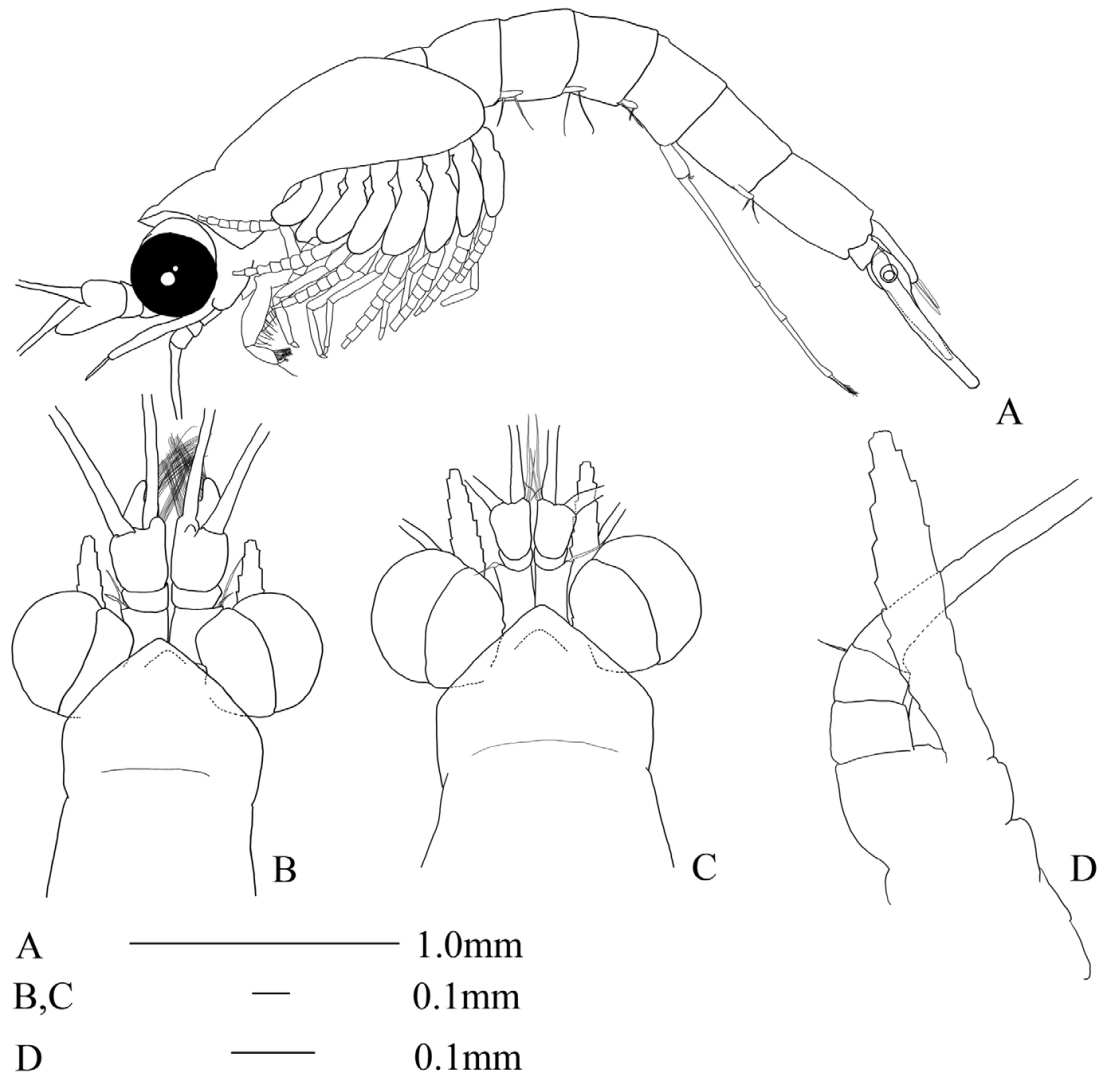
Anisomysis (Anisomysis) phuketensis sp. n., **A, B, D** holotype **C** allotype **A** lateral view **B** anterior part of body **C** anterior part of body **D** antenna.

Body slender (Fig. 5A). Carapace extending anteriorly into obtusely triangular rostrum with bluntly pointed apex, covering bases of antennules (Fig. 5B, C).

Eyes large, cornea occupying half of eye in dorsal view (Fig. 5A–C). Eyestalk very short, without papilliform process on dorsal surface.

Antennular peduncle more robust in male (Fig. 5B) than that in female (Fig. 5C); first segment shorter than third, armed with two setae at anterolateral corner; second segment shortest. In female (Fig. 5C), first segment armed with single seta at anterolateral corner.

Antennal scale (Fig. 5D) closely near the anterior margin of antennular peduncle in male (Fig. 5B), well beyond anterior margin in female (Fig. 5C); 5.5 times as long as broad in male, 6.1 times as long as broad in female. Antennal peduncle short, not reaching middle of antennal scale in both sexes (Fig. 5D).

**Figure 6.**
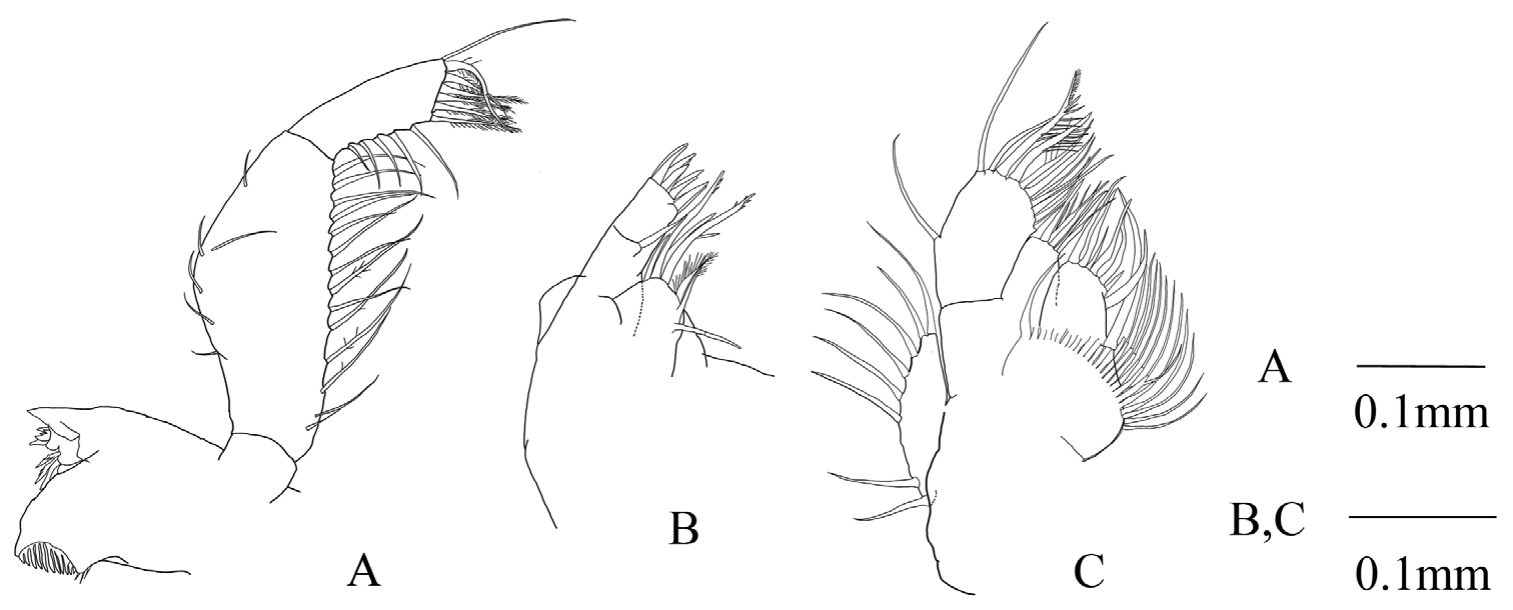
Anisomysis (Anisomysis) phuketensis sp. n., **A–C** holotype **A** mandible and mandibular palp (right side) **B** maxillule **C** maxilla.

Mandibular palp (Fig. 6A) 3-segmented; second segment widened mesially at around mid-length, armed with setae on both margins, without triangular processes; third segment rectangular, 0.5 times as long as second segment, outer margin armed with 5 marginal setae increasing in length distally, distal margin with 5 barbed setae on margin, 1 recurved and barbed seta and 1 long seta at distomedial corner. Maxillule and maxilla as shown in Fig. 6B and C, respectively.

**Figure 7.**
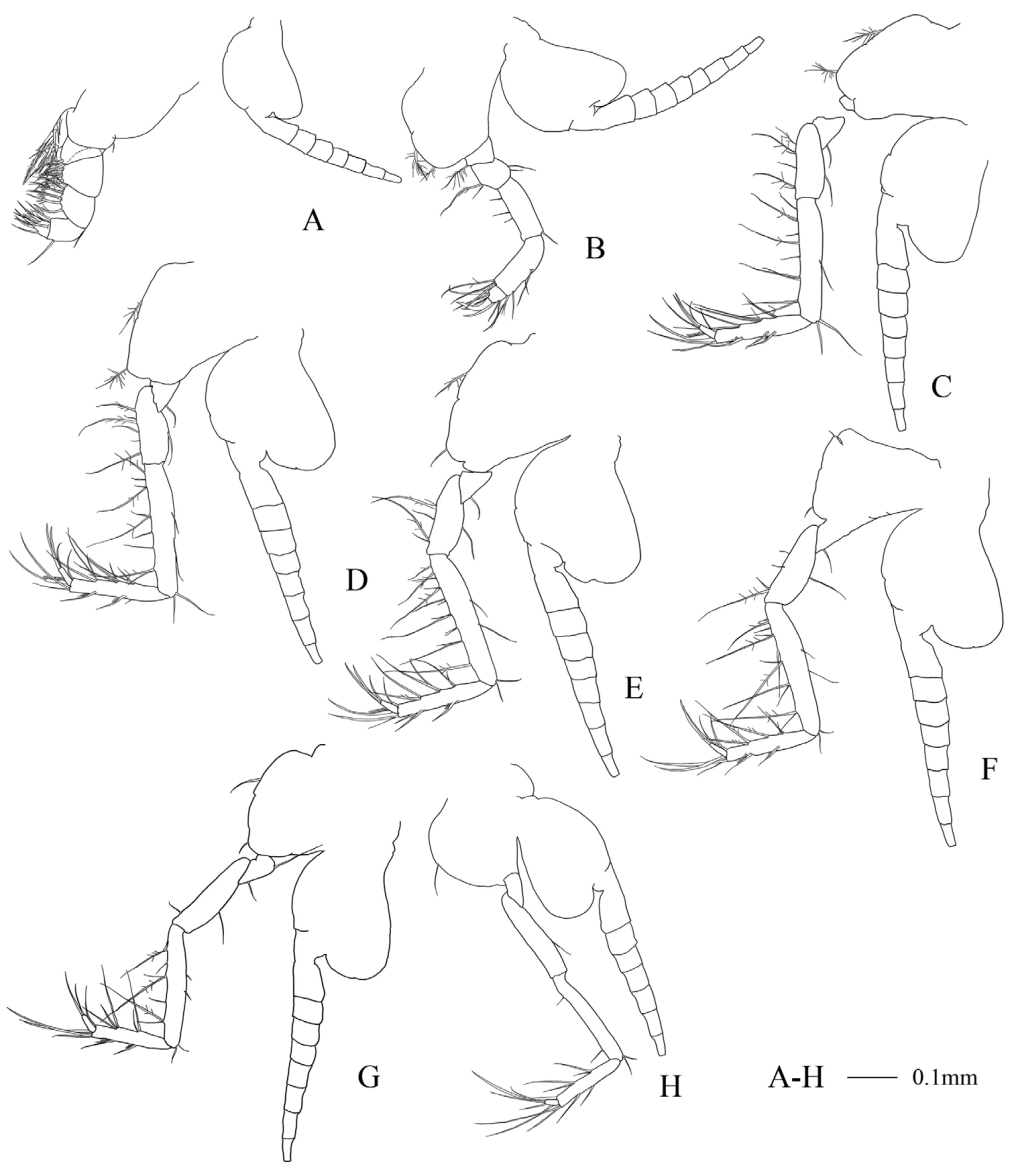
Anisomysis (Anisomysis) phuketensis sp. n., **A–H** holotype **A** 1^st^ thoracopod **B** 2^nd^ thoracopod **C** 3^rd^ thoracopod **D** 4^th^ thoracopod **E** 5^th^ thoracopod **F** 6^th^ thoracopod **G** 7^th^ thoracopod **H** 8^th^ thoracopod.

First thoracopodal endopod (Fig. 7A) short and stout, armed with straight, strong terminal claw. Second thoracopodal endopod (Fig. 7B) short; merus as long as carpopropodus, dactylus with strong, curved terminal claw. Third to eighth thoracopodal endopods (Fig. 7C–H) with undivided carpopropodus in both sexes. Flagelliform part of first and eighth thoracopodal exopods 7-segmented (Fig. 7A, H) and second to seventh 8-segmented (Fig. 7B–G). Basal plate of eight thoracopodal exopods with rounded outer distal corners.

Abdomen (Fig. 5A) long and slender, sixth somite 1.3 times longer than fifth.

**Figure 8.**
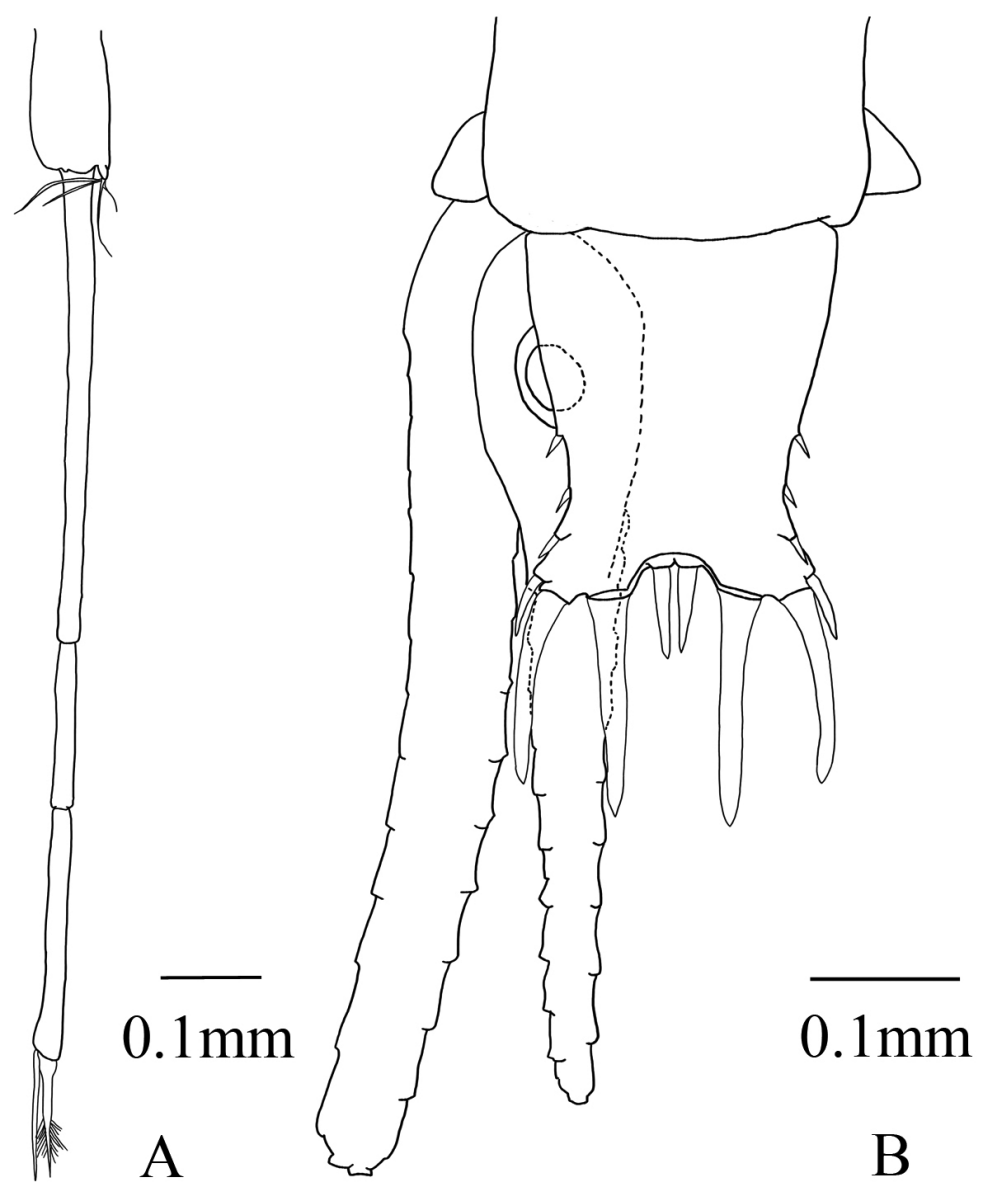
Anisomysis (Anisomysis) phuketensis sp. n., **A, B** holotype **A** 4^th^ pleopod **B** uropods and telson

First, second, third, and fifth pleopods of male and all pleopods of female rudimentary. Fourth male pleopod (Fig. 8A) biramous; endopod minute and thin lobe with 4 setae; exopod long, three-segmented, overreaching distal end of telson (Fig. 5A). First segment longer than second and third segments combined; second segment shortest; segment length ratios 3:1:1.5; third segment with two terminal setae, almost equal in length, outer setae slender and naked, inner setae swollen at base and barbed on distal half.

Uropod slender, setose around (Fig. 8B); endopod straight, 1.5 times longer than telson excluding apical spines, no spine in statocyst region; exopod slightly curved outward, 1.1 times as long as endopod.

Telson (Fig. 8B) short, nearly 3/5 length of sixth abdominal somite, 1.2 times longer than broad at base, compressed around distal 1/4, then expanding distally, distal margin slightly narrower than base; lateral margin armed with 4-5 short spines; apex of telson concave at the middle with paired spines almost equal in length, apical margin truncate with two long stout spines, outer spine curved inward, slightly shorter than inner straight spine.

##### Etymology.

The species is named after the type locality.

##### Remarks.

The most noticeable characteristic of Anisomysis (Anisomysis) phuketensis is the form of the telson. This species resembles Anisomysis (Anisomysis) robustispina Panampunnayil, 1984 and Anisomysis (Anisomysis) truncata Panampunnayil, 1993 in the presence of the peculiar long stout spines on the apical margin of telson. However, Anisomysis (Anisomysis) phuketensis is distinguished from Anisomysis (Anisomysis) robustispina by the following characters: only two long stout spines on telson (three in the latter), the length/width ratio of telson being 1.2 (1.6 in the latter), the length ratio of uropodal endopod to telson being 1.5 (2.3 in the latter). Although the telson of Anisomysis (Anisomysis) truncata is also armed with two pairs of stout apical spines, the outer spines are twice as long as the inner (subequal in Anisomysis (Anisomysis) phuketensis) and lacks the medial depression with two small spines, which is present in Anisomysis (Anisomysis) phuketensis and Anisomysis (Anisomysis) robustispina.

##### Distribution.

The type locality and Ko Chueak, Hat Chao Mai National Park, Trang Province, Thailand.

### Key to the subgenera of the genus *Anisomysis* (cited from Murano and Fukuoka 2003)

**Table:** 

1	Body rather strongly built, gibbous; abdomen flexed ventrally; eye large, with cornea divided into two parts by groove	***Pseudanisomysis* Murano & Fukuoka, 2003**
–	Body slender, straight; eye globular, expanded, not divided into two portions	**2**
2	Mandibular palp with second segment armed with triangular processes on mesial margin	***Paranisomysis* Băsescu, 1973**
–	Mandibular palp with second segment armed with normal setae on both margins	**3**
3	Carapace with spinules on antero-lateral margin; telson with un-articulated denticles on lateral margin	***Javanisomysis* Băcescu, 1992**
–	Carapace without spinules on antero-lateral margin; telson with articulated denticles on lateral margin	***Anisomysis* Băcescu, 1973**

### Key to the species of the subgenus *Anisomysis*

#### Subgenus *Anisomysis* Băsescu, 1973

**Type species.** *Anisomysis laticauda* Hansen, 1910.

**Description.** Body straight, slender, not hispid. Cornea of eye large, globular, not divided into two portions. Antennular peduncle having neither expanded lobe nor finger-like process on second segment. Second segment of mandibular palp foliate, without triangular denticles on mesial margin. Telson variable with basally articulated denticles on lateral margin.

**Table:** 

1	Telson longer than last abdominal somite	***Anisomysis sirielloides* Băsescu, 1975**
–	Telson shorter than last abdominal somite	**2**
2	Telson without distal cleft	**3**
–	Telson with distal cleft	**11**
3	Telson triangular with narrow apex	**4**
–	Telson with rounded or truncate distal margin	**5**
4	Rostrum triangular with narrowly rounded apex; exopod of fourth male pleopod with second segment 1/3 as long as third segment; marginal spines of telson increasing distally in length, apical spine 3 times as long as broad at base	***Anisomysis mixta* Nakazawa, 1910**
–	Rostrum broadly rounded or triangular with broadly rounded apex; exopod of fourth male pleopod with second segment about 4/5 as long as third segment; marginal spines of telson subequal in length, apical spine 1.5 times as long as broad at bas	***Anisomysis australis* Zimmer, 1918**
5	Distal margin of telson rounded	**6**
–	Distal margin of telson truncate or weakly truncate	**7**
6	Telson 1.5 times as long as broad, with 10–12 spines on posterior half of each lateral margin	***Anisomysis chessi* Murano, 1983**
–	Telson nearly twice as long as broad, with 7–8 spines on posterior 2/3 of each lateral margin	***Anisomysis quadrispinosa* Wang, 1989**
7	Telson with constriction, more than 10 spines on each lateral margin	***Anisomysis enewetakensis* Murano, 1983**
–	Telson without constriction	**8**
8	Telson armed with 4–5 spines on each lateral margin	**9**
–	Telson armed with 9–13 spines on each lateral margin	**10**
9	Telson rounded triangular with weakly truncate distal margin; distal spines of telson subequal in size	***Anisomysis levi* Băsescu, 1973**
–	Telson trapezoid with truncate distal margin; distal spines of telson longer and stouter than lateral spines	***Anisomysis truncata* Panampunnayil, 1993**
10	Each lateral margin of telson with 9 spines. Exopod of fourth male pleopod reaching tip of telson	***Anisomysis bacescui* Pillai, 1976**
–	Each lateral margin of telson with 10–13 spines. Exopod of fourth male pleopod reaching beyond base of telson	***Anisomysis comorensis* Wooldridge & Mees, 2004**
11	Inner margin of telson cleft unarmed with spines	**12**
–	Inner margin of telson cleft armed with spines	**22**
12	Uropodal endopod with process on mesial margin of statocyst region	**13**
–	Uropodal endopod without process on mesial margin of statocyst region	**14**
13	Process on uropodal endopod blunt, without articulation at base	***Anisomysis bifurcata* Tattersall, 1912**
–	Process on uropodal endopod acutely pointed, with articulation at base	***Anisomysis spinata* Panampunnayil, 1993**
14	Each apical lobe of telson with single spine	**15**
–	Each apical lobe of telson with 2 or 3 spines	**19**
15	Telson cleft about half of telson length	**16**
–	Telson cleft less than 1/3 of telson length	**17**
16	Rostrum pointed; eyestalk with papilliform process; telson with 2 or 3 spines on lateral margin of each posterior lobe	***Anisomysis megalops* (Illig, 1913)**
–	Rostrum rounded; eyestalk without papilliform process; telson with 5 or 6 spines on lateral margin of each posterior lobe	***Anisomysis nana* Murano, 1995**
17	Each lateral margin of telson with 11–20 spines	***Anisomysis minuta* Liu & Wang, 1983**
–	Each lateral margin of telson with less than 10 spines	**18**
18	Each lateral margin of telson with 5–9 short slender spines. Cleft of telson 1/3 length of telson	***Anisomysis pelewensis* Ii, 1964**
–	Each lateral margin of telson with 3 small spines. Cleft of telson 1/5 length of telson	***Anisomysis unispinosa* Wooldridge & Mees, 2004**
19	Telson narrowing abruptly at distal 1/3, each lateral margin with 2 spines at narrow part; each apical lobe of telson with 2 short spines	***Anisomysis kunduchiana* Băsescu, 1975**
–	Telson gradually narrowing, each lateral margin with more than 4 spines; each apical lobe of telson with 2 or 3 spines	**20**
20	Telson with V-shaped cleft, each lateral margin with 7–11 spines; each apical lobe of telson with 2 spines	***Anisomysis hawaiiensis* Murano, 1995**
–	Telson with U-shaped cleft, each lateral margin with 4–7 spines	**21**
21	Each apical lobe of telson with 3 spines, each lateral margin armed with 6 or 7 spines	***Anisomysis incisa* Tattersall, 1936**
–	Each apical lobe of telson with 2 spines, each lateral margin armed with 4 to 6 spines	***Anisomysis pescaprae* Connell, 2009**
22	Posterior margin of telson narrow; each apical lobe of telson with 1 spine	***Anisomysis extranea* Murano, 1995**
–	Posterior margin of telson broad; each apical lobe of telson with more than 3 spines	**23**
23	Distal margin of telson with median depression, armed with more than 4 spines	**24**
–	Distal margin of telson with slight median sinus, armed with 2 spines	**30**
24	Bottom of telson cleft convexed	**25**
–	Bottom of telson cleft rounded	**26**
25	Telson 1.3 times as long as broad. Exopod of fourth male pleopod not extending beyond anterior margin of telson	***Anisomysis hanseni* Nouvel, 1967**
–	Telson 1.5 times as long as broad. Exopod of fourth male pleopod extending to distal end of telson	***Anisomysis mullini* Murano, 1987**
26	Telson cleft with bottom spines only	***Anisomysis neptuni* Connell, 2009**
–	Telson cleft with spines entirely covered	**27**
27	Exopod of fourth male pleopod extending to anterior margin of last abdominal somite	**28**
–	Exopod of fourth male pleopod extending to or beyond posterior margin of last abdominal somite	**29**
28	Apical cleft as long as 1/9 of telson, each lateral margin of telson with 3 or 4 spines	***Anisomysis aikawai* Ii, 1964**
–	Apical cleft as long as 2/5 of telson, each lateral margin of telson with 5 or 6 spines	***Anisomysis spinaintus* sp. n.**
29	Exopod of fourth male pleopod extending to middle of telson, second segment 1.6 times longer than third	***Anisomysis hashizumei* Fukuoka & Murano, 1997**
–	Exopod of fourth male pleopod extending slightly beyond anterior margin of telson, second segment slightly longer than third	***Anisomysis laticauda* Hansen, 1910**
30	Distal margin of telson armed with 2 or 3 pairs of long and robust spines	31
–	Distal margin of telson without long and robust spines	**32**
31	Posterior margin of telson broader than basal width, with 3 pairs of long and robust spines, about 1/3 of telson length	***Anisomysis robustispina* Panampunnayil, 1984**
–	Posterior margin of telson equal to or narrower than basal width, with 2 pairs of long and robust spines, about 2/5 of telson length	***Anisomysis phuketensis* sp. n.**
32	Telson 1.3–1.4 times as long as broad	**33**
–	Telson 1.5–1.8 times as long as broad	**35**
33	Posterior 2/3 of telson gradually narrowing distally; lateral spines of telson considerably reduced in size	***Anisomysis vasseuri* Ledoyer, 1974**
–	Posterior 1/4 to 1/3 of telson almost parallel-sided; lateral spines of telson normally developed	**34**
34	Rostrum broadly rounded; uropodal endopod subequal to exopod in length; length ratios of 3 exopod segments of fourth male pleopod 3.1 : 1 : 1.5	***Anisomysis rotunda* Murano & Fukuoka, 2003**
–	Rostrum triangular with rounded apex; uropodal endopod clearly shorter than exopod; length ratios of 3 exopod segments of fourth male pleopod 5.5 : 1 : 2.6	***Anisomysis maldivensis* Murano & Fukuoka, 2003**
35	Lateral spines of telson considerably reduced in size	**36**
–	Lateral spines of telson normally developed	**37**
36	Antennal scale not extending to distal end of antennular peduncle in male, slightly beyond in female, 6 times as long as broad; telson 1.5 times as long as broad, with 7 spines on each lateral margin	***Anisomysis boraboraensis* Murano, 1995**
–	Antennal scale extending beyond distal end of antennular peduncle in both sexes, 7 times as long as broad; telson 1.7 times as long as broad, with 8 or 9 spines on each lateral margin	***Anisomysis parvispina* Murano & Fukuoka, 2003**
37	Lateral depression of telson clear at distal quarter; distal margin of telson broad, with 4 or 5 pairs of long spines	***Anisomysis brevicauda* Wang, 1989**
–	Lateral depression of telson very slight if present; distal margin of telson narrow, with 3 pairs of long spines	***Anisomysis akajimaensis* Murano, 1990**

### Note about the subgenus *Pseudanisomysis*

In the middle of September 2015, the subgenus Pseudanisomysis is treated as a junior synonym of the subgenus Carnegieomysis in the World Register of Marine Species (WoRMS: Mees, 2015). The reference is Mees J (2015) Anisomysis (Carnegieomysis) W. Tattersall, 1943. In: Mees J, Meland K (Eds.) World List of Lophogastrida, Stygiomysida and Mysida. Accessed through: World Register of Marine Species at http://www.marinespecies.org/aphia.php?p=taxdetails&id=456543 on 2015–09–29.

## Supplementary Material

XML Treatment for Anisomysis (Anisomysis) spinaintus

XML Treatment for Anisomysis (Anisomysis) phuketensis
